# T_H_9, T_H_17, and T_H_22 Cell Subsets and Their Main Cytokine Products in the Pathogenesis of Colorectal Cancer

**DOI:** 10.3389/fonc.2019.01002

**Published:** 2019-10-04

**Authors:** Guanglin Cui

**Affiliations:** ^1^Research Group of Gastrointestinal Diseases, The Second Affiliated Hospital of Zhengzhou University, Zhengzhou, China; ^2^Faculty of Health Science, Nord University, Levanger, Norway

**Keywords:** colorectum, tumorigenesis, cytokine, T_H_, therapeutic

## Abstract

In recent years, several newly identified T helper (T_H_) cell subsets, such as T_H_9, T_H_17, and T_H_22 cells, and their respective cytokine products, IL-9, IL-17, and IL-22, have been reported to play critical roles in the development of chronic inflammation in the colorectum. Since chronic inflammation is a potent driving force for the development of human colorectal cancer (CRC), the contributions of T_H_9/IL-9, T_H_17/IL-17, and T_H_22/IL-22 in the pathogenesis of CRC have recently become an increasingly popular area of scientific investigation. Extensive laboratory and clinical evidence suggests a positive relationship between these new T_H_ subsets and the growth and formation of CRC, whereas, administration of IL-9, IL-17, and IL-22 signaling inhibitors can significantly alter the formation of colorectal chronic inflammation or CRC lesions in animal models, suggesting that blocking these cytokine signals might represent promising immunotherapeutic strategies. This review summarizes recent findings and currently available data for understanding the vital role and therapeutic significance of T_H_9/IL-9, T_H_17/IL-17, and T_H_22/IL-22 in the development of colorectal tumorigenesis.

## Introduction

Colorectal cancer (CRC) is one of the leading causes of high cancer-associated mortality worldwide. According to the colorectal adenoma-carcinoma sequence theory, most CRCs develop from pre-existing adenomatous lesions through a multistep process with the accumulation of genetic, molecular and histological changes (1, 2). This process is significantly influenced by host immunity (3–5). T-helper (T_H_) 1 immunity is believed to be the most important host immune mechanism for keeping pre-neoplastic/neoplastic cells in check (6–8). To escape host immune surveillance, however, pre-neoplastic adenoma cells can develop diverse strategies to suppress antitumor immunity.

Studies in recent years have suggested that diverse T_H_ subsets play a crucial role in the processing and maintenance of chronic inflammation (9) and tumorigenesis (10), in which their cytokine products play an essential role that modulates the development of the inflammatory microenvironment provoked by tumorigenesis and affect tumor initiation/progression (3, 11, 12). Therefore, there is great interest in studying the role of T_H_ subsets and their cytokine products in CRC initiation/progression. Indeed, many reports have observed notably altered immune cell densities and cytokine profiles during CRC development (13–21), in which significantly increased CD4 and CD8-positive T lymphocyte populations are observed (22–24), along with decreased expression levels of T_H_1 cytokines and increased expression levels of T_H_2 cytokines in patients with CRC (7, 8, 25, 26). Recent studies have also revealed that altered cytokine profiles are correlated with disease stage and prognosis in patients with CRC (25, 27–29). Furthermore, both *in vivo* and *in vitro* studies have demonstrated that blocking cytokine signaling, as seen in interleukin (IL)-6 and IL-17 studies, significantly suppresses cell growth, proliferation, progression, and metastasis in CRC (21, 30). Therefore, ample evidence suggests that cytokines are not only key contributors to the pathogenesis of CRC (18, 20, 31–34) but also potential immunotherapeutic targets for the treatment and prevention of CRC (35, 36).

Recently, several newly identified T_H_ subsets, for example, T_H_9, T_H_17, and T_H_22, have been reported to be associated with the development of chronic inflammation and carcinogenesis. The cytokine products of these subsets, IL-9, IL-17, and IL-22, respectively, are potent proinflammatory mediators and greatly contribute to the initiation and maintenance of colorectal inflammatory disorders (9, 37–39). Emerging evidence suggests that these cytokines are also involved in the development and progression of CRC, as administration of cytokine inhibitors significantly suppressed the development of colorectal inflammation and subsequent CRC occurrence in mice. Therefore, an improved understanding of the immunopathogenesis of T_H_ subsets and their cytokine products will be helpful for designing novel translational/targeted approaches to CRC (40).

In view of the importance and increasing data about T_H_9, T_H_17, and T_H_22 cells and their associated cytokines in the initiation and development of CRC, here, we review recent findings, with an emphasis on the function of T_H_9/IL-9, T_H_17/IL-17, and T_H_22/IL-22 in the pathogenesis of CRCs, and discuss the therapeutic significance of these main cytokines in the prophylaxis of CRC.

## Tumor-Infiltrating Lymphocytes (TILs) and Chromosomal Instability in Patients With CRC

TILs in colorectal adenoma/CRC have been intensively studied. The results suggest that significantly increased densities of TILs are observed in both the tumor stroma and adenoma/CRC epithelium (41, 42), and have clinical significance in predicting prognosis and chemotherapeutic outcomes in patients with CRC (43–46). Phenotypic analysis revealed that these TILs are mostly CD4-positive and CD8-positive cells. CD4-positive TILs are predominantly T_H_ cells localized within the tumor stroma, and CD8-positive TILs are cytotoxic T lymphocytes (CTLs) that infiltrate both the tumor stroma and adenoma/CRC epithelium (23, 47–50). In addition, clinical molecular analysis revealed that ~85% of CRCs may present with chromosomal instability, while 15% display DNA mismatch repair (MMR) (51, 52). Therefore, another issue worthy of consideration is the influence of DNA microsatellite instability (MSI) on the presentation pattern of lymphocyte infiltration in CRC. Indeed, Boissière-Michot et al. (53) have reported that CRCs with MSI exhibited increased densities of CD3-positive, CD8-positive, CD45RO-positive, and T-bet-positive TILs compared with those of microsatellite stable (MSS) CRCs, whereas, the density of FoxP3-positive regulatory T cells (Tregs) was not significantly different between the two groups (53). Lee et al. examined TILs in CRCs with MSI and found that CD8-positive TILs, but not CD4-positive TILs, were significantly increased in both tumor cell niches and the tumor stroma in CRCs with MSI (54). Similarly, Matsutani et al. (23) recently showed that the density of CD8-positive TILs is associated with MMR status in CRC and intratumor CD8-positive TILs in the invading margins in randomly selected fields, and tends to be higher in MMR-deficient patients than in MMR-proficient patients (23). More recently, several studies have shown that chromosomal instability patterns significantly influence the infiltration of CD8-positive lymphocytes, survival, and therapeutic response in patients with CRC (55–57).

Collectively, these results demonstrate that chromosomal instability status in patients with CRC may significantly influence TIL infiltration.

## The Role of T_H_9/IL-9, T_H_17/IL-17, and T_H_22/IL-22 in the Development of CRC

### T_H_9/IL-9

T_H_9, a novel subset of T_H_ cells that primarily produce IL-9 (58), has recently been shown to be involved in the pathogenesis of inflammatory bowel disease (IBD) (59, 60). The production of IL-9 by T_H_ cells is stimulated by IL-4, IL-21, and transforming growth factor (TGF)-β and inhibited by interferon (IFN)-γ (61). Transcriptional studies have revealed that the production of IL-9 by T_H_9 cells is regulated by PU.1 (an ETS family transcription factor) (62), and there is ample evidence to suggest that the T_H_9 cytokine IL-9 could be a strong proinflammatory factor in the induction of experimental colitis (38, 63–65). An animal study showed that T_H_9 cells labeled with PU.1 were markedly increased in the inflamed mucosa (lamina propria) of dextran sulfate sodium (DSS)-induced colitis mice (66). In human ulcerative colitis (UC) specimens, we found that increased populations of PU.1-positive T_H_9 cells were predominantly located in the lamina propria, whereas, intraepithelial T_H_9 cells were present at very low densities and were only occasionally observed (66). However, it is worth noting that PU.1 is not a lineage-specific transcription factor for IL-9 induction in T_H_9 cells, as it can also be expressed in other subsets under certain circumstances. For example, it has been reported that PU.1 is expressed in B cells (67). In addition to PU.1, ETV5, another ETS family member, is an essential transcriptional factor for T_H_9 cells, as deficiency of both ETV5 and PU.1 in T cells results in markedly reduced induction of IL-9 (68). Therefore, a range of transcription factors might be involved in the differentiation of T_H_9 cells (69). Furthermore, a number of clinical studies have revealed that serum levels of IL-9 in patients with UC or Crohn's disease (CD) are higher than those in controls, and increased levels of IL-9 are significantly associated with inflammatory activity in the bowel in patients with UC or CD (37, 70, 71). Tian et al. showed that the involvement of IL-9 in the pathogenesis of UC occurs through activation of the Stat3/SOCS3 signaling pathway (72). These data support the notion that the activation of the T_H_9 subset and its cytokine product, IL-9, participates in the regulation of inflammatory processes in the bowel. Since chronic inflammation, as seen in IBD, may predispose patients to CRC development, the role of the proinflammatory cytokine IL-9 in the development of CRC has begun to attract increasing attention. Indeed, published studies have explored the role of IL-9 in different types of cancers, and both antitumor and protumor effects have been reported (73–75). Rivera Vargas et al. demonstrated that autophagy controls IL-9 secretion by T_H_9 cells and that selective autophagy targets PU.1 for degradation in CD4 T cells, resulting in the negative regulation of T_H_9 homeostasis and antitumor immunity (73, 76). Two clinical studies performed in southern China reported that expression levels of IL-9, in both the plasma and CRC tissues, are lower in patients with CRC compared to levels in healthy controls and are significantly associated with TNM staging. CRC patients with advanced stages have reduced levels of IL-9 compared to those with early stages (77, 78). Liu et al. demonstrated that IL-9 induces the conversion of Tregs to T_H_9 cells, subsequently inhibiting tumor growth in the CT26 colon cell line (79). Wang et al. reported a similar finding in Chinese patients with CRC. They found that the expression levels of IL-9 protein and messenger RNA were lower than those in the controls and correlated with TNM staging, Dukes staging, lymph node metastasis, and good prognosis. *In vivo*, IL-9 overexpression significantly inhibited tumor growth and resulted in a longer survival time in a mouse subcutaneous allograft model (80). Moreover, an ectopically expressed membrane-bound form of IL-9 induces an immunostimulatory effect that suppresses the growth of CT26 colon cancer cells (81). These studies seem to suggest that T_H_9 cells have an antitumor effect that largely depends on their ability to secrete IL-9 and may influence tumor growth by enhancing immune responses (73).

In contrast to the above findings, other studies have described a protumor effect of IL-9. We have more recently demonstrated that a population of IL-9-positive cells and protein expression levels of IL-9 are greatly increased in mouse colitis-associated cancer (CAC) tissues, compared to those of control mouse tissues (82). Tian et al. examined the expression levels of IL-9 in 12 pairs of CAC tissues and adjacent non-tumor tissues by immunohistochemistry, and found that the expression of IL-9 in CAC tissues was markedly higher than that in adjacent tissues (83). *In vivo*, this study showed that IL-9 overexpression in RKO and Caco-2 colon cancer cell lines results in an increasing proliferation rate that occurs through upregulating expression of c-Myc and cyclin D1 (83). Furthermore, a study from Poland demonstrated that serum levels of IL-9 in 104 CRC patients were significantly higher than those in controls (84), in contrast to reports from southern China (77, 78). Hoelzinger et al. recently used IL-9-deficient mice to investigate the effects of IL-9 in multiple models of breast and colon cancer development. These authors found that eliminating endogenous IL-9 enabled sensitization of host T cells to tumors, leading to early rejection without the requirement for vaccines or immunomodulatory therapies. Notably, IL-9-deficient mice acquired immunologic memory, which actively protected them from residual disease and tumor re-challenge, an effect that is linked to the activation of CD8-positive T cells (74).

**Figure 1:**
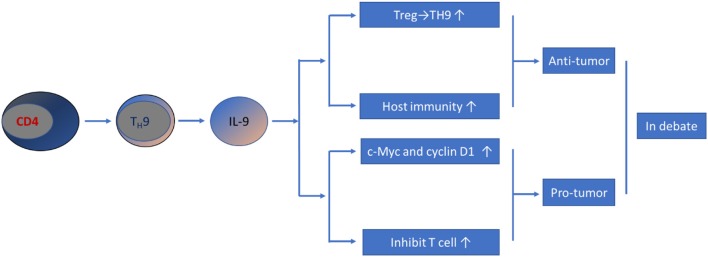
Schematic representation of T_H_9/IL-9's role in CRC pathogenesis. Current data have yielded controversial results; both pro-tumor and anti-tumor effect have been reported.

Collectively, the current literature has revealed that T_H_9/IL-9 exhibit both antitumor and protumor effects (Figure 1). These controversial results could be due to the distinct experimental models, techniques and tumorigenesis conditions used in different studies. Moreover, contradictory data may also stem from differential effects of IL-9 on immune (antitumor) vs. cancer (protumor) cells. Therefore, the effect of T_H_9/IL-9 in the development of CRC is still an issue of debate, and much work remains to be done.

### T_H_17/IL-17

The IL-17-expressing T_H_ cell subset (known as T_H_17 cells) is another T_H_ cell subset related to colorectal inflammation and tumorigenesis (39). Studies suggest that the differentiation of T_H_17 cells is stimulated by a set of upstream cytokines, including IL-1β, IL-6, IL-21, IL-23, and TGF-β, and the main cytokine products of this cell type are IL-17A and IL-17F (39). It should be mentioned that the intestine is a site of origin for T_H_17 cells, and whether they function as non-pathogenic T_H_17 cells promoting homeostasis or pathogenic T_H_17 cells involved in infection and autoimmunity is dependent on the upstream stimulator. TGF-β stimulation skews the differentiation of T_H_17 cells to a non-pathogenic phenotype that promotes homeostasis, and IL-23 accelerates the differentiation of T_H_17 cells toward a pathogenic phenotype that is involved infection and autoimmunity. Some previous studies have shown an antitumor effect of IL-17. For instance, Kryczek et al. reported that subcutaneous injection of the MC38 murine colon cancer cell line into IL-17-deficient mice resulted in faster growth and increased lung metastasis foci compared to that of wildtype control mice (85). Previous studies have demonstrated that the antitumor effect of IL-17 in various cancer types is related to enhanced recruitment and activity of lymphocytes, natural killer (NK) cells, and DCs into the tumor site and production of the antitumor cytokine IFN-γ (86–89). Lin et al. showed that CRC patients with higher IL-17 levels tend to have a better prognosis than those with lower IL-17 levels (90). However, the majority of published studies suggest that T_H_17/IL-17 has profound effects on triggering and amplifying the inflammatory process and tumorigenesis in the colorectum (21, 91, 92). Considerable evidence suggests that increased expression profiles of the T_H_17 cytokine product, IL-17A, are associated with the production of the proangiogenic factor vascular endothelial growth factor (VEGF) and poor prognosis in patients with CRC (93). The profile of circulating and intra-tumoral T_H_17 cells in patients with colon cancer, with respect to tumor staging, was analyzed. The results revealed that the percentage of T_H_17 cells in both the peripheral blood and cancer tissues, assessed by multicolor flow cytometry, was significantly increased in cancer patients compared to that of healthy controls or adjacent non-tumor tissues, respectively. In addition, the percentage of T_H_17 cells in both the peripheral blood and cancer tissues was associated with disease stage, and it was higher in patients with advanced stages than in those with early stages (94). Furthermore, functional studies identified that the type A receptor (IL-17RA) is a critical mediator of IL-17 in promoting colorectal tumorigenesis (95). We have, for the first time, examined IL-17A dynamics along the human colorectal adenoma-carcinoma spectrum (31) and have found that the expression of IL-17A, at both the mRNA and protein levels, was significantly increased in the adenoma stage and persisted to the CRC stage. Since the differentiation of T_H_17 cells is stimulated by a set of cytokines, IL-1β and IL-6, which are important for early stages, and IL-23, which is important for late stages of development and the expansion of T_H_17 cells, we examined these stimulating factors. The data showed that T_H_17 differentiation-stimulating factors IL-1β, IL-6, and IL-23, but not TGF-β, were significantly increased in a similar expression pattern as IL-17 along the colorectal adenoma-carcinoma sequence, suggesting activated T_H_17 differentiation and elevated IL-17 production throughout this sequence. Interestingly, we found that increased IL-17 expression levels was associated with an increased dysplasia grading score in adenomas, which is one of the most important histological parameters in assessing the neoplastic progression of an adenoma toward a CRC. Analysis of IL-17A gene variants revealed that heterozygous rs10484879 was associated with increased disease risk, while the rs3748067 genotype was associated with a reduced risk of CRC (96). Samiei et al. revealed that the IL-17A197AA genotype was significantly associated with an increased CRC risk in Malaysian patients, compared with those with the GG and AG genotypes (97). These data suggest that IL-17A increases susceptibility to CRC. More recently, the contribution of IL-17 to chemotherapeutic agent resistance in CRC has been investigated. Cisplatin-based anticancer therapy is an important chemotherapeutic strategy for the treatment of CRC. Sharp et al. reported that elevated systemic levels of the T_H_17 cytokines, IL-17F and IL-23, were associated with stage IV colon cancer (98). Furthermore, the effect of IL-17 on the induction of chemoresistance in CRC is mediated through targeting phosphorylated protein kinase B (p-Akt), the apoptosis regulators Bax and Bcl-2 and the serine/threonine-protein kinase mTOR (99).

Despite general advances in diagnosis and treatment, the long-term survival of CRC remains low, except when early curative surgery is performed. The prognostic significance of T_H_17/IL-17 in human CRC has been evaluated. Le Gouvello et al. have shown that increased expression of IL-17 in CRC tumor tissues is associated with proficient MMR status, indicating poor prognosis in patients with CRC (100). Other studies have also confirmed that increased levels of IL-17 are associated with aggressive features and poor prognosis in CRC (93, 101). Moreover, a clinical study revealed that CRC patients with high expression of the T_H_17 cluster and a high density of IL-17-positive cells *in situ* may have a shorter disease-free survival time and early relapse after primary CRC tumor surgical resection (24). Since systemic metastasis has often already occurred at diagnosis, approximately half of CRC patients ultimately die due to relapse and metastasis after resection of primary tumors (102). Therefore, recent studies have explored the influence of T_H_17/IL-17 on CRC metastasis. The promoting effect of IL-17 on colorectal tumorigenesis has been reported in mouse CRC models (103). The data showed that the development of CRC is facilitated via the activation of T_H_17 cells, whereas, blocking IL-17A signaling significantly suppressed hyperplastic and neoplastic lesion formation (103), suggesting that activation of T_H_17/IL-17 plays an important role in the formation of premalignant lesions and the establishment of CRC lesions. Thus, targeting T_H_17/IL-17 signaling may represent a promising approach for future CRC immunotherapy (104).

In addition to IL-17A, IL-17F has been shown to be another potential factor involved in the development of CRC. Tong et al. have shown a protective effect of IL-17F in the development of CRC. They found decreased tumor growth of IL-17F-transfected HCT116 cells compared to that of mock transfectants when transplanted into nude mice. However, increased colonic tumor numbers and tumor areas were detected in IL-17F-deficient mice compared to those of wildtype mice after colon cancer induction (105). Furthermore, Nemati et al. found that the IL-17F T7488 allele is associated with a decreased risk of CRC and tumor progression, whereas, the AG genotype of the IL-17A G197A SNP is associated with an increased risk of CRC (106). However, Chae and Bothwell reported that IL-17F deficiency significantly inhibits spontaneous intestinal tumorigenesis in the small intestine of Apc(Min/+) mice, probably through decreased IL-1β, Cox-2, and IL-17 receptor C (IL-17RC) expression and decreased infiltration of immune cells in the lamina propria (107). The role of IL-22 in the pathogenesis of CRC is also somewhat controversial (see T_H_22/IL-22 section). In addition, T_H_17 cells under certain circumstances can coproduce IL-9, IFN-γ, or IL-10. The function of these cytokines in the development of CRC might be significantly different. Finally, some T_H_17 cytokines can be made by many T_H_ subsets, and T_H_17 differentiation-stimulating factors may also regulate other T_H_ subsets under certain conditions. This makes the cytokine network in human tumors very complex.

**Figure 2:**
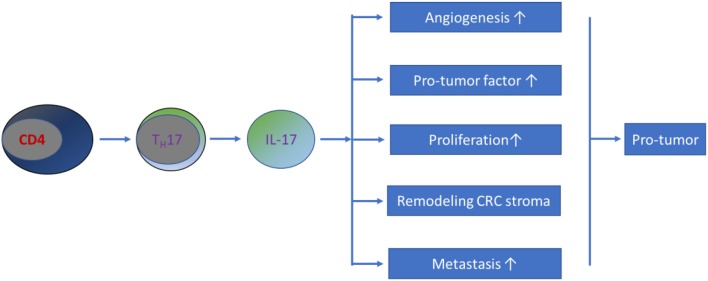
Schematic representation of T_H_17/IL-17's role in CRC pathogenesis. Most current studies have demonstrated that T_H_17/IL-17 exhibits a pro-tumor effect on the development of CRC through a mixture of mechanisms.

In summary, current evidence has shown that IL-17 promotes metastasis in CRC. Possible mechanisms by which this occurs include the following: (1) IL-17 stimulates the activation of metastasis-related genes, such as tumor necrosis factor-α (TNF-α), nuclear factor kappa B (NF-κB), CC chemokine receptor 6 (CCR6), matrix metalloproteinase (MMP)-2, MMP-7, MMP-9, and MMP-13 (108–111). (2) IL-17 potentially enhances angiogenesis by increasing the production of VEGF (93, 104). Further studies revealed that IL-17 does not directly stimulate the growth of vascular endothelial cells but strongly induces the migration and cord formation of endothelial cells (92, 112). (3) IL-17 is capable of remodeling the CRC stroma (113). (4) IL-17 promotes the production of myeloid-derived suppressor cells that create a favorable milieu for CRC development and progression (114–117). Therefore, the involvement of T_H_17/IL-17 in the pathogenesis of CRC occurs through a mixed pathway (Figure 2).

### T_H_22/IL-22

T_H_22 cells compose another novel T cell subset. RORγt acts as a positive transcription factor and T-bet acts as a negative transcription factor for T_H_22 cell differentiation (118). In addition, AhR has also been considered a transcription factor for IL-22 produced by T_H_22 cells (119). T_H_22 cells produce cytokines, such as IL-22, IL-26, and IL-33. Although it is not clear whether T_H_22 cells are a separate subset or derived from T_H_17 cells, given that there is still debate about whether IL-22 is also a T_H_17 cytokine, many observations obtained over the past few years have indicated that numerous factors, including IL-1β, IL-6, IL-21, and IL-23, may stimulate the production of IL-22 in activated T_H_22 cells (118, 119). The role of IL-22 in modulating the progression of inflammation, as seen in IBD, has been primarily studied (120, 121). However, these results are somewhat controversial. One study reported that UC patients exhibited significantly reduced numbers of IL-22-positive cells in actively inflamed tissues, compared with the numbers in both non-inflamed tissues and normal tissues from healthy controls (9). Another study conducted in patients with CD reported that IL-22 protects the intestinal barrier by enhancing intestinal epithelial cell migration in CD (122), which was supported by the findings that anti-TNF-α therapy in patients with CD results in increased IL-22 production by CD4-positive T cells and enhanced repair of damaged mucosa (123). Sugimoto et al. reported that IL-22 gene delivery elicited rapid attenuation of local intestinal inflammation by enhancing Stat3 activation within colonic epithelial cells in mice (124). However, other studies have shown the opposite relationship between T_H_22/IL-22 and chronic inflammation in the colorectum. Analysis of IL-22 levels and circulating T_H_22 cells in UC showed that UC patients had higher levels of IL-22 and T_H_22 cells than those of healthy controls, and both features were associated with disease severity (125, 126). In addition, IL-22 antibody administration significantly reduced colorectal inflammation in DSS-induced colitis mice compared with that of control mice (127). Therefore, current data from most studies suggest that T_H_22/IL-22 might participate in the development and modulation of colorectal inflammation.

**Figure 3:**
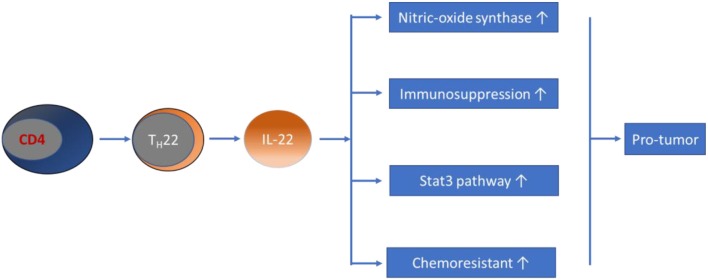
Schematic representation of T_H_22/IL-22's role in CRC pathogenesis. Evidence obtained from most studies has suggested that T_H_22/IL-22 promotes the development of CRC through diverse pathways.

Regarding the role of T_H_22/IL-22 in cancers, evidence from early studies revealed that IL-22 promotes the growth of tumor cells in many types of cancers, including lung adenocarcinoma and hepatocellular carcinoma (128, 129). In patients with gastric cancer, dysregulation of circulating T_H_22 cell frequency and IL-22 expression levels have been demonstrated, and were associated with advanced tumor features and patient survival (130, 131). Regarding the role of T_H_22/IL-22 in CRC, *in vitro* studies have shown that IL-22 enhances nitric oxide synthase expression in colon cancer cells, significantly contributing to conversion to nitrites, which are associated with colonic inflammation and carcinogenesis (132). Subsequent studies have shown that IL-22 has a direct proliferative effect on colonic epithelial cells (133). Sun et al. revealed that coculture of single cells, isolated from colon cancer tissues with recombinant IL-22 for 24 h, resulted in a significantly increased proliferation rate, which could be blocked by an anti-IL-22 antibody (134). Furthermore, IL-22 potentially stimulates intestinal epithelial cells to secrete IL-10, a main contributor to the formation of an immunosuppressive milieu in CRC (135). IL-22 has also consistently been shown to be protumor in CRC animal models. The results obtained from both *in vivo* and *in vitro* models illustrated that IL-22 is associated with CRC progression through activating the Stat3 pathway (133, 134, 136, 137). Various studies in recent years have attempted to clarify the promoting effect of IL-22 in the pathogenesis of CRC. The results showed that BALB/c nude mice transplanted subcutaneously with RKO colon cancer cells tended to have faster tumor growth after intraperitoneal injection of IL-22 every other day, compared to that of control mice (136). Recently, Wang et al. revealed that IL-22 is associated with the development of dysplasia in a murine model of CAC (138). By using IL-22BP-deficient mice, Huber et al. showed that the soluble receptor of IL-22, IL-22 binding protein (IL-22BP), has a crucial role in controlling tumorigenesis and epithelial cell proliferation in the mouse colon (133). In support of the hypothesis that T_H_22 is involved in the development of CRC, Doulabi et al. reported that increased populations of intratumoral T_H_17, T_H_22, and CD4-positive T cells, coproducing IL-17/IL-22 in patients with colon cancer, were observed in tumor tissues (94). This result is in contrast with the results of a previous study that reported markedly reduced percentages of T_H_22 cells and IL-22 levels in CRC patients, compared with those of healthy controls that were negatively associated with the pathological stages of CRC (139). Another clinical study demonstrated that the percentage of T_H_22 cells in the CD4-positive T cell subset was significantly higher in CRC tissues, compared to that of para-tumor tissues, as determined by flow cytometry in patients with CRC (136). In addition, IL-22 genetic polymorphisms have been shown to be a risk factor for colon cancer (140), and elevated serum IL-22 levels correlate with chemoresistance in patients with CRC (141). Furthermore, both IL-22 and its functional transmembrane receptor (IL-22R1) have been found to be significantly expressed in CRC epithelial and tumor stromal cells (142). Taken together with the above findings, most current data suggest a promoting effect of T_H_22/IL-22 on the development of CRC (see Figure 3), making it an attractive target for anticancer therapy.

### Potential Therapeutic Significance of T_H_9/IL-9, T_H_17/IL-17, and T_H_22/IL-22 in CRC

Because of the current lack of effective immunotherapies for the treatment of CRC, the search for novel immunotherapeutic targets has become critically important (36, 143). Strong evidence now suggests that chronic inflammation is a potent driving force for tumor development, in which diverse T_H_ cells and their cytokine products play essential roles (144, 145). It is therefore hypothesized that T_H_ cells and their cytokine network may represent potential therapeutic targets for the treatment of human cancer (14, 146–151). Immunotherapy against various cancer types, including CRC, has been well-reviewed (146, 152). These outstanding reviews have indicated that current immunotherapy strategies, including anti-cytokine antibodies, immune adjuvants, and vaccines, immune checkpoint inhibitors and immune cell-based therapies, can potentially enhance host antitumor immune function and prolong patient survival time in human cancers (152, 153).

As the above findings indicated that T_H_9 and its cytokine product IL-9 are associated with the development of chronic inflammation in the colorectum, additional studies further analyzed the therapeutic effect of blocking IL-9 signaling in a colitis mouse model (66). We were able to show that blocking IL-9 signaling by intraperitoneal administration of IL-9 antibody rapidly ameliorates colorectal inflammation in DSS-induced colitis mice (66). However, the role of T_H_9/IL-9 in the development of CRC remains unclear. To the best of our knowledge, studies examining the therapeutic effect of blocking IL-9 signaling in the development of CRC remains to be investigated. Furthermore, several studies have looked at the therapeutic effect of blocking IL-17A in the treatment and prevention of CRC (92, 110–112). Numasaki et al. have shown that IL-17 promotes tumor cell growth *in vivo* by enhancing angiogenesis in mice with MCA205/IL-17 tumors, while angiogenic activity was markedly suppressed by a neutralizing IL-17 monoclonal antibody (112). We previously demonstrated that administration of an IL-17A antibody strongly suppresses the development of DSS/DMH-induced CRC in mice (20). These findings have highlighted a key role of IL-17 in CRC development. Currently, secukinumab, ixekizumab, and brodalumab, which are biological agents that target IL-17 signaling, are being studied and have successfully passed phase III clinical trials for rheumatoid arthritis and psoriatic arthritis. These antibodies may represent new options for potential CRC treatments; therefore, it would be interesting to evaluate the therapeutic effect of these antibodies alone or combined with chemotherapy in treating relapsed or metastatic CRCs (104). Since the T_H_22-related cytokine IL-22 has been reported to promote tumorigenesis in distinct cancer entities, including lung, ovarian, breast, gastric, pancreatic, and colon cancers, the IL-22-IL-22R1 pathway has become an attractive target for anticancer therapy (154–156). An examination of the role of IL-22 in lung adenocarcinoma has shown that IL-22 in K-ras-driven lung cancer mediates tumor proliferation and remodeling of the tumor microenvironment in a mouse model (129). Since a previous study demonstrated that IL-22 has a crucial role in controlling tumorigenesis and epithelial cell proliferation in the colon (133), IL-22 may represent an attractive therapeutic target. Sun et al. examined the effect of IL-22 on the proliferation of single cells from colon cancer tissues by coculture with or without recombinant IL-22 for 24 h. The results showed that recombinant IL-22 stimulates colon cell proliferation, while anti-IL-22 blocks this effect (134). Another study tested the efficacy of a novel IL-22BP-based colon cancer gene therapy strategy (157). By delivering the IL-22BP gene with a newly developed non-viral gene vector, DOTAP-modified mPEG-PCL micelles (DMP), the inhibitory efficacy of the DMP/IL-22BP complex on mouse colon cancer was examined via intraperitoneal administration. These results showed that the DMP/IL-22BP complex blocked IL-22 signaling and exhibited a significant inhibitory effect on cancer cell growth both *in vitro* and *in vivo*. These anticancer mechanisms may be related to induction of apoptosis, enhanced infiltration of lymphocytes in tumor tissues and inhibition of angiogenesis. The results suggest that blocking IL-22 signaling may represent a promising strategy for CRC cancer gene therapy (157). Administration of a specific antibody is one of the simplest and most efficient methods to block cytokine signaling, and one study demonstrated that antibody-based targeted delivery against IL-22 in DSS-induced colitis mice resulted in more rapid recovery from clinical symptoms and improved colon morphology compared to controls (127). Currently, the delivery of IL-22 antibodies in a CRC animal model has not been tested, and evaluation of therapeutic efficacy remains unknown.

## Concluding Remarks

Current findings suggest a pivotal role of the T_H_ subsets, T_H_17 and T_H_22, in the pathogenesis of CRC. In this context, their cytokine products have been identified as key contributors in CRC tumorigenesis through several pathways, such as stimulating proliferation, enhancing angiogenesis, recruiting immune suppressor cells, and remodeling tumor stroma. Therefore, strategies aimed at suppressing these cytokine signals as a new immunotherapeutic option for the treatment and prevention of CRC are of key interest. Regarding the role of T_H_9 and its cytokine product IL-9 in the pathogenesis of CRC, it is rather diverse as it can have a protumor function as well as antitumor characteristics. Therefore, the contribution of T_H_9/IL-9 in CRC development remains an issue of debate. It is hoped that further studies will eventually elucidate the precise mechanisms and molecular characterization of signaling pathways involved in T_H_ subset cytokine actions in CRC, which will in turn help to improve immunotherapeutic approaches.

## Author Contributions

The author confirms being the sole contributor of this work and has approved it for publication.

### Conflict of Interest

The author declares that the research was conducted in the absence of any commercial or financial relationships that could be construed as a potential conflict of interest.

## Abbreviations

CAC: colitis associated cancer
CCR: CC chemokine receptor
CD: Crohn's disease
CRC: colorectal cancer
DMH: 1,2-dimethylhydrazine
DMP: the DOTAP modified mPEG-PCL micelles
DSS: dextran sulfate sodium
IBD: inflammatory bowel disease
IFN-γ: interferon γ
IL: interleukin
IL-17RA: IL-17 type A receptor
IL-22BP: IL-22 binding protein
MMP: matrix metalloproteinase
MMR: mismatch repair
MSI: microsatellite instable
NF-κB: nuclear factor kappa B
p-Akt: phosphorylated-protein kinase B
TGF-β: transforming growth factor β
T_H_: T helper
TIL: tumor-infiltrating lymphocyte
TNF-α: tumor necrosis factor α
Treg: regulatory T cell
UC: ulcerative colitis
VEGF: vascular endothelial growth factor.
